# Supplementary material for: The impact of deep learning reconstruction in low dose computed tomography on the evaluation of interstitial lung disease
Source: PLoS One. 2023 Sep 27;18(9):e0291745. doi: 10.1371/journal.pone.0291745 (PMC10529569; doi:10.1371/journal.pone.0291745)

**Deep learning reconstruction model**

In our study, ClariCT.AI version 1.2 was used, which received clearance from the Korean medical device registration and approval (version 1.0 has received U.S Food and Drug Administration clearance, version 1.1 has received Conformité Européenne certification). DLMs are built upon a modified U-Net architecture, which is a special subtype of convolutional neural network. A U-Net with five layers of convolutional filtering for contracting and expanding paths was built based on optimization experiments using different combinations of structures. (sFigure 1).

The DLM was trained by using a noise-added CT image as an input to produce an original CT image as an output (sFigure 2). It was trained with diverse vendor-specific LDCT images from different vendors to acquire generalized learning and vendor-agnostic denoising capability. A synthetic sinogram-based LDCT simulation technique was used to generate a paired set of low-dose and standard-dose CT images from a set of standard-dose CT images [1].

The training dataset consisted of more than one million CT images encompassing 2,100 different combinations of scan and reconstruction conditions, including varying kVp, mAs, automatic exposure control, slice thickness, contrast enhancement, and convolution kernels with 24 scanner models from four different CT manufacturers (GE Healthcare, Siemens, Philips Healthcare, and Cannon); 80% of the dataset was used for model training, while the remaining 20% was used for validation. The trained deep learning model could be used for denoising both FBP and IR CT images, regardless of the CT vendor and reconstruction type. The performance of the DLM has been previously evaluated in several clinical studies [2-9].

To select the optimal denoising option for chest CT in this study, we considered combinations of denoising options, including denoising strength and sharpness enhancement. After evaluation with a pilot image set, we selected a noise blending value of 0 and the edge blending value was set to 0.

1. Ahn C, Heo C, Kim JH. Combined low-dose simulation and deep learning for CT denoising: application in ultra-low-dose chest CT. In:*International Forum on Medical Imaging in Asia 2019*: International Society for Optics and Photonics, 2019; 110500E

2. Lee S, Choi YH, Cho YJ, Lee SB, Cheon J-E, Kim WS, et al. Noise reduction approach in pediatric abdominal CT combining deep learning and dual-energy technique. *European Radiology* 2021;31:2218-2226

3. Hong JH, Park E-A, Lee W, Ahn C, Kim J-H. Incremental image noise reduction in coronary CT angiography using a deep learning-based technique with iterative reconstruction. *Korean journal of radiology* 2020;21:1165

4. Kolb M, Storz C, Kim JH, Weiss J, Afat S, Nikolaou K, et al. Effect of a novel denoising technique on image quality and diagnostic accuracy in low-dose CT in patients with suspected appendicitis. *European journal of radiology* 2019;116:198-204

5. Lim WH, Choi YH, Park JE, Cho YJ, Lee S, Cheon J-E, et al. Application of vendor-neutral iterative reconstruction technique to pediatric abdominal computed tomography. *Korean journal of radiology* 2019;20:1358-1367

6. Nam JG, Ahn C, Choi H, Hong W, Park J, Kim JH, et al. Image quality of ultralow-dose chest CT using deep learning techniques: potential superiority of vendor-agnostic post-processing over vendor-specific techniques. *European radiology* 2021:1-9

7. Yeoh H, Hong SH, Ahn C, Choi J-Y, Chae H-D, Yoo HJ, et al. Deep Learning Algorithm for Simultaneous Noise Reduction and Edge Sharpening in Low-Dose CT Images: A Pilot Study Using Lumbar Spine CT. *Korean journal of radiology* 2021;22:1850

8. Choi H, Chang W, Kim JH, Ahn C, Lee H, Kim HY, et al. Dose reduction potential of vendor-agnostic deep learning model in comparison with deep learning–based image reconstruction algorithm on CT: A phantom study. *European radiology* 2021:1-9

9. Park S, Yoon JH, Joo I, Yu MH, Kim JH, Park J, et al. Image quality in liver CT: low-dose deep learning vs standard-dose model-based iterative reconstructions. *European radiology* 2021:1-10

**S1 Fig. Network architecture of the modified U-net built for deep learning models**

Deep learning models are built on a modified U-Net architecture, which is a special subtype of convolutional neural network. The U-Net shown here with five layers of convolutional filtering for contracting and expanding paths was built based on optimization experiments using different combinations of structures


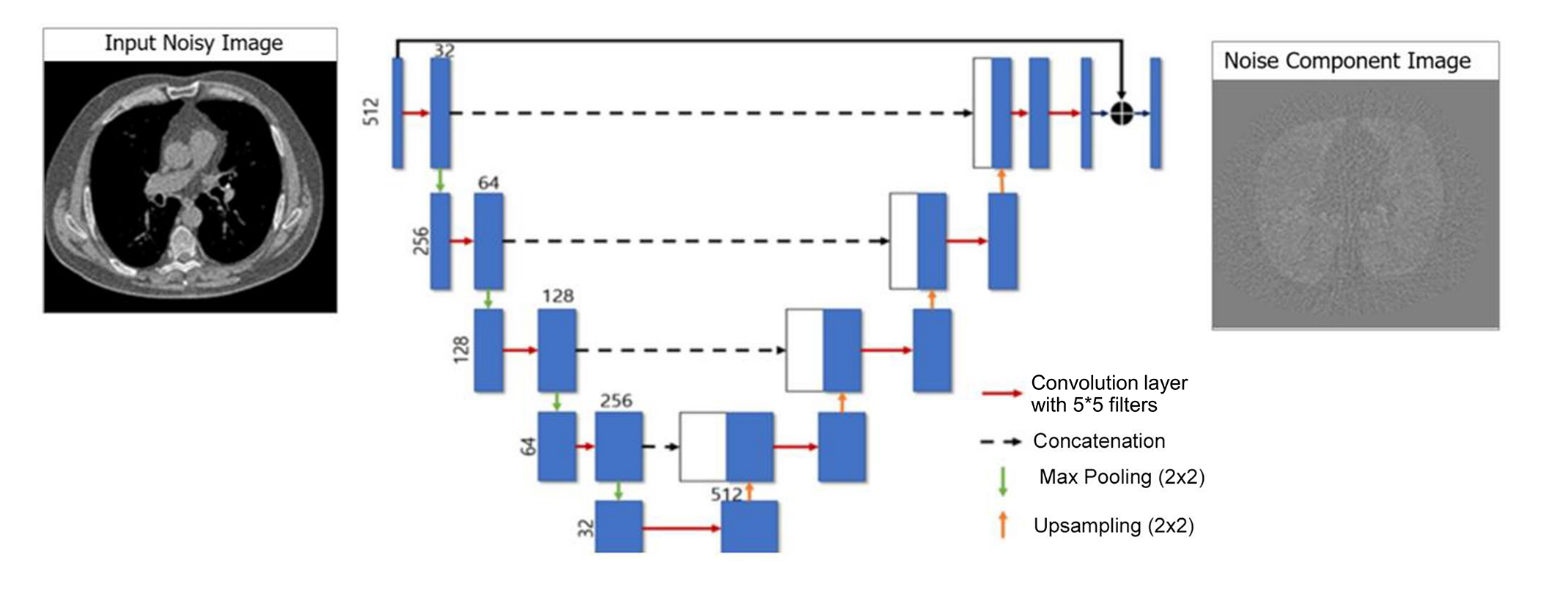


**S2 Fig. Diagram of proposed deep learning imaging reconstruction method**

A low-dose computed tomography (CT) image is transferred from the CT scanner to the digital imaging and communications in medicine (DICOM) receiver module through the DICOM storage service class provider protocol of the DICOM 3.0 communication standard. Then, the ‘Denoiser’ module performs noise reduction on the received CT image. Finally, ‘DICOM Sender’ module transfers the denoised image to the picture archiving and communication system (PACS) system through DICOM storage service class user protocol.


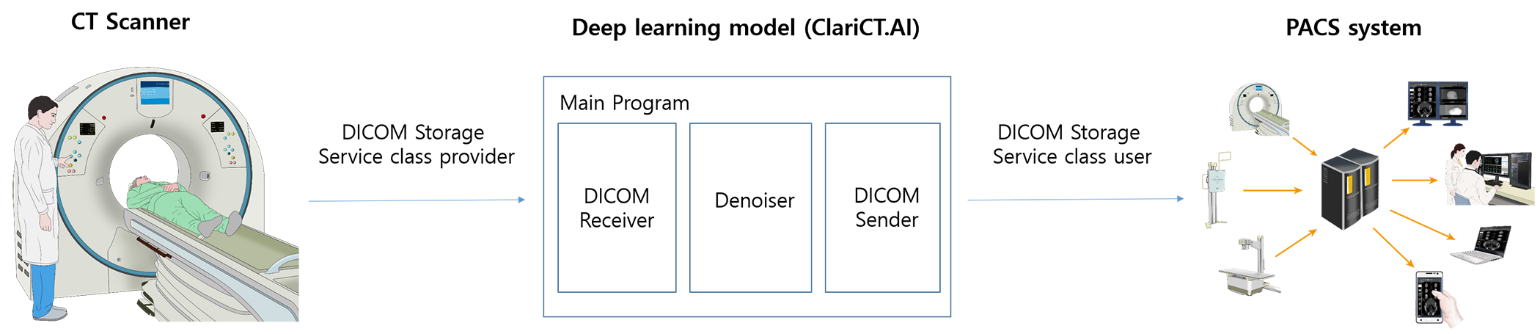

Supplement: S1 File — (DOCX) [file pone.0291745.s001.docx]
