# Supplementary material for: The impact of deep learning reconstruction in low dose computed tomography on the evaluation of interstitial lung disease
Source: PLoS One. 2023 Sep 27;18(9):e0291745. doi: 10.1371/journal.pone.0291745 (PMC10529569; doi:10.1371/journal.pone.0291745)
Supplement: Attachment — Submitted filename: Response to reviewer 20230803.docx [file pone.0291745.s003.docx]

Dear. Professor Zhentian Wang

Academic editor of PLOS ONE journal

Thank you for inviting us to re-submit a revised draft of our manuscript. We also appreciate the time and effort you and each of the reviewers have dedicated to providing insightful feedback on ways to strengthen our paper. We have incorporated changes that reflect the detailed suggestions you have graciously provided.

To facilitate your review of our revisions, the following is a point-by-point response to the questions and comments delivered in the website.

#1. Conclusion states "The diagnostic efficacy was maintained in the CT pattern diagnosis of UIP in DLM".
What was the study data supporting this conclusion? Authors only presented the agreement of the diagnostic categorization of IPF between the three reconstruction methods (FBP, IR, DLM), while none of which is appropriate for reference standard.

- Thanks for the good point. As you pointed out, since the reference standard is not clear enough to draw such a conclusion, we have deleted the content and simply state the agreement between different reconstruction methods.

#2 How was the reference standard established for diagnostic categorization ?
In Lines 303-304, reference standard was made by a consensus panel of two radiologist, which varied depending upon recon methods. And Lines 195-198 state that those readers depended on HRCT findings of the patient to make a reference standard. These are apparently discrepant and cause a confusion.

- Sorry for the confusion. For discrepant cases, two radiologists referred to HRCT and made a consensus opinion. We will fix that part clearly. (Page 9, Lines 194-200)

3. While Lines 331-332 states "There were no discrepant cases in the alternative diagnoses between the three reconstruction methods.", the next sentence (Lines 332-333) says "Cases with discrepant diagnoses among the three reconstruction methods are presented in Table 4." Which one is correct? Again, question arises "What was the main question of the study? Was it evaluating the diagnostic efficacy? Or simply finding agreement between different reconstructions?"

- I am sorry that this part has confused you. The ‘alternative diagnosis’ refers to one of the four classifications of CT pattern diagnosis of UIP. ‘Discrepant diagnosis’ literally refers to cases in which different diagnoses were made between observers. We edited that part to make it more clear. (Page 15, Lines 333-334)
- As you pointed out, since the reference standard is not clear enough to draw such a conclusion, we will delete ‘diagnostic efficacy’ part and simply state the agreement between different reconstruction methods.

4. Figures 3-5 were used to show the discordant cases between FBP, IR, DLM, and HRCT. How the findings in these Figures relate to study purpose and conclusion ? These Figures seem to convey a contradictory message to the conclusion stating "The diagnostic efficacy was maintained in the CT pattern diagnosis of UIP in DLM".

- Among the four classifications of UIP CT pattern diagnosis, in the case of UIP pattern, all but one discrepant case showed a discrepant opinion. In addition, in the case of 'alternative diagnosis', which requires a completely different diagnosis than IPF, 100% concordant diagnosis was shown.
- It is noteworthy that the agreement was almost perfect in these two classifications, since when UIP pattern is radiologically diagnosed in CT, it is sufficient to secure a diagnosis without lung biopsy in an appropriate clinical contest. Discrepant case was found between FBP and other two reconstruction method, and we think that it was result due to too much noise due to the nature of the FBP image (actually, for this reason, the FBP image is seldom used in clinical practice and considered as RAW images when reconstructing CT images).
- In other words, most discrepant cases showed differences between ‘probable UIP’ and ‘indeterminate UIP’. It is difficult to make a definitive diagnosis with CT alone in these two categories, and additional biopsies or multidisciplinary approaches are needed in most cases.
- We have embedded this in discussion part. Thank you (Page 21-22, Lines 460-473)

5. Lines 302-303 states that there was a substantial agreement between the two readers (κ = 0.617). Was it a pooled agreement for the three recons? Was there a better agreement with DLM compared to other recons ?

- No. We used the average kappa in our analysis. We have addressed it in metod section (Page 10, Line 206). The agreement between each reconstruction was not calculated separately.

6. Table 3 compares the diagnostic categorization among the three recon methods without using HRCT, whereas in Table 4 HRCT was used (as a reference ?) along with recon methods. Why were two different ways used ?

- Table 3 shows the overall diagnosis distribution, and Table 4 was added to describe discrepant cases in more detail.

Also 'Final diagnosis' appears only in Table 4. Does it mean there was no final diagnosis for the rest 186 cases ? If it was true, then what was reference standard for them and how the diagnostic efficacy was made ?

- The final diagnosis of the patients are described in the baseline characteristics in result section. (Page 10-11, Lines 221-233)
- A total of 93 patients (48.2%) were diagnosed with IPF based on the diagnostic criteria of the American Thoracic Society and European Respiratory Society [29], 55 patients (28.5%) were diagnosed with connective tissue disease related ILD, and 19 patients (9.8%) were diagnosed with interstitial lung abnormality. Organizing pneumonia was diagnosed in six patients (3.1%), followed by smoking related ILD in six (3.1%), nonspecific interstitial pneumonia in four (2.1%), and pleuroparenchymal fibroelastosis in four patients (2.1%). Remaining six patients (3.1%) had other diagnoses (for example, chronic hypersensitivity pneumonitis, post inflammatory fibrosis, sarcoidosis, hemosiderosis). Lung biopsy was performed on 38 patients, either wedge resection (34 patients) or transbronchial lung biopsy (4 patients). Of these, 17 were pathologically confirmed to have UIP on surgical lung biopsy.
- IPF is a clinical diagnosis, and the final diagnosis is made through a multidisciplinary approach in addition to CT finding.

7. PLOS authors have the option to publish the peer review history of their article ([what does this mean?](https://journals.plos.org/plosone/s/editorial-and-peer-review-process#loc-peer-review-history)). If published, this will include your full peer review and any attached files.

- We only follow the journal's publication policy. If you do not want your comments reflected in the publication, we will request the journal to keep them private.
